# Supplementary material for: Enhancing Lung Cancer Classification through Integration of Liquid Biopsy Multi-Omics Data with Machine Learning Techniques
Source: Cancers (Basel). 2023 Sep 14;15(18):4556. doi: 10.3390/cancers15184556 (PMC10526503; doi:10.3390/cancers15184556)
Supplement: Supplementary file 1 [file cancers-15-04556-s001.zip › cancers-2578605-supplementary.pdf]

Supplementary Figures

Enhancing Lung Cancer Classification Through Integration of Liquid Biopsy Multi-Omics Data with Machine Learning Techniques

Hyuk-Jung Kwon[1,2†], Ui-Hyun Park[1†], Chul Jun Goh[1], Dabin Park[1], Yu Gyeong Lim[1], Isaac Kise Lee[1,2,3], Woo-Jung Do[1], Kyoung Joo Lee[1], Hyojung Kim[1], Seon-Young Yun[1], Joungsu Joo[1], Na Young Min[1], Sunghoon Lee[1], Sang-Won Um[4], Min-Seob Lee[1,5*]

[1] R&D Department, Eone-Diagnomics Genome Center, Inc., 291 Harmony-ro, Yeonsu-gu, Incheon, 22014, Republic of Korea

[2] Department of Computer Science and Engineering, Incheon National University (INU), Incheon, 22012, South Korea,

[3]NGENI Foundation, California, USA

[4]Division of Pulmonary and Critical Care Medicine, Department of Medicine, Samsung Medical Center, Sungkyunkwan University School of Medicine, 81 Irwon-ro, Gangnam-gu, Seoul, 06351, Republic of Korea

[5]Diagnomics, Inc., 5795 Kearny Villa Rd. San Diego, CA 92123, USA

[†]These authors contributed equally

*Correspondence: Min Seob Lee, Ph.D.

Eone-Diagnomics Genome Center, Inc., 291 Harmony-ro, Yeonsu-gu, Incheon, 22014, Republic of Korea

Phone No: +82-10-3080-1393

Email: mlee@edgc.com

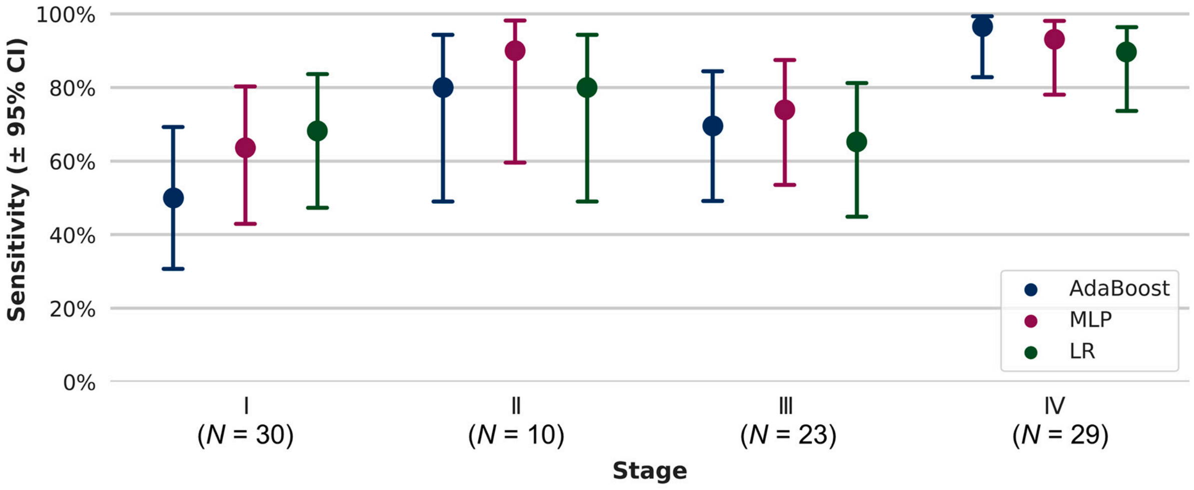

Supplementary Figure S1. Sensitivity plot representing stage-wise cancer detection performance of each machine learning model. The sensitivity values of the three machine learning models, AdaBoost (blue dots), MLP (dark red dots), and LR (dark green dots), are presented using a dot plot. The x-axis shows the stages of lung cancer and 'N' indicates the number of patients. The y-axis represents the sensitivity values. The lines extending above and below each dot represent the 95% confidence interval for that value, considering a specificity of 90%.

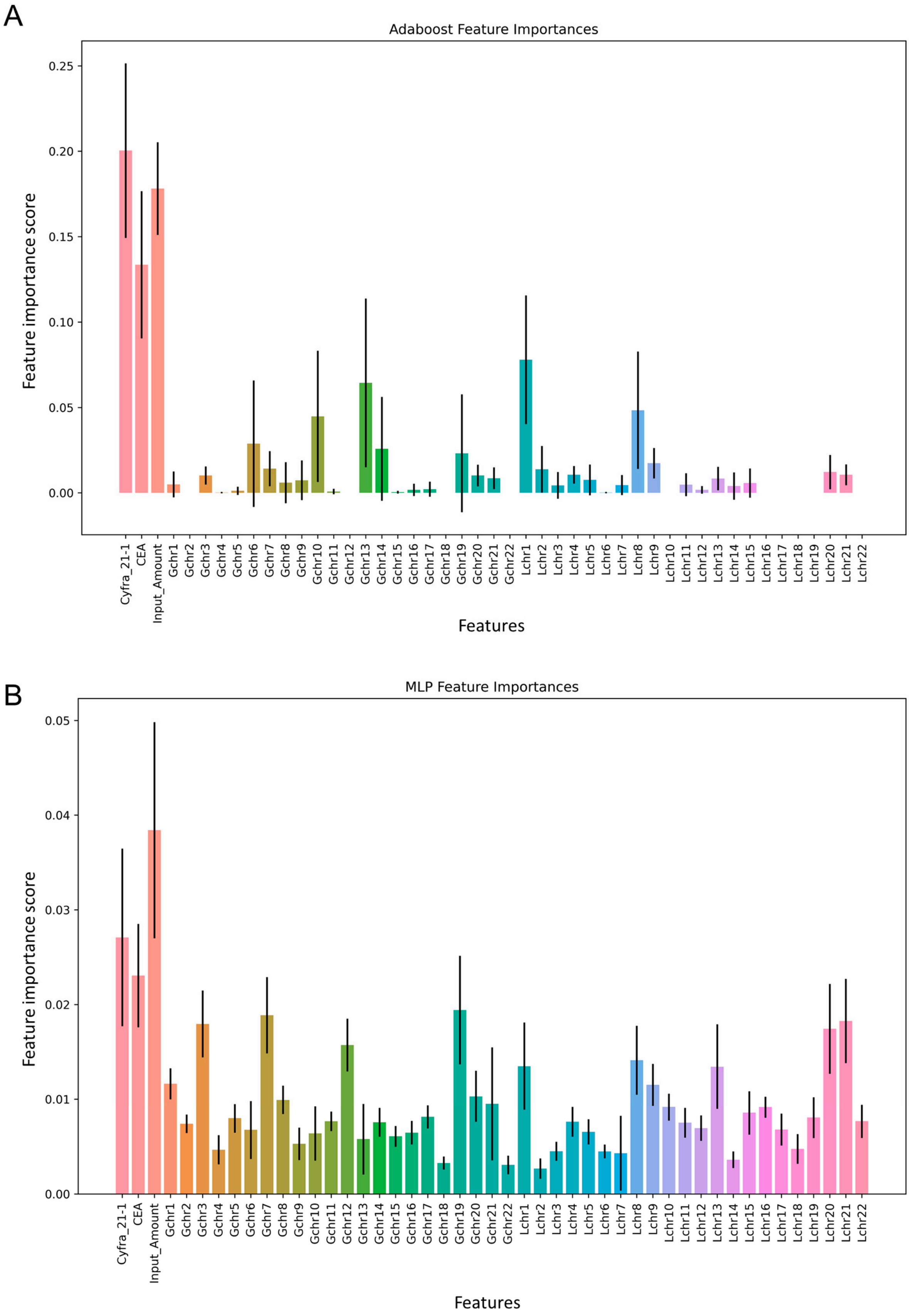
A
Adaboost Feature Importances
Feature importance score
0.25
0.20
0.15
0.10
0.05
0.00
Cyfra_21-1
CEA
Input_Amount
Gchr1
Gchr2
Gchr3
Gchr4
Gchr5
Gchr6
Gchr7
Gchr8
Gchr9
Gchr10
Gchr11
Gchr12
Gchr13
Gchr14
Gchr15
Gchr16
Gchr17
Gchr18
Gchr19
Gchr20
Gchr21
Gchr22
Lchr1
Lchr2
Lchr3
Lchr4
Lchr5
Lchr6
Lchr7
Lchr8
Lchr9
Lchr10
Lchr11
Lchr12
Lchr13
Lchr14
Lchr15
Lchr16
Lchr17
Lchr18
Lchr19
Lchr20
Lchr21
Lchr22
Features
B
MLP Feature Importances
Feature importance score
0.05
0.04
0.03
0.02
0.01
0.00
Cyfra_21-1
CEA
Input_Amount
Gchr1
Gchr2
Gchr3
Gchr4
Gchr5
Gchr6
Gchr7
Gchr8
Gchr9
Gchr10
Gchr11
Gchr12
Gchr13
Gchr14
Gchr15
Gchr16
Gchr17
Gchr18
Gchr19
Gchr20
Gchr21
Gchr22
Lchr1
Lchr2
Lchr3
Lchr4
Lchr5
Lchr6
Lchr7
Lchr8
Lchr9
Lchr10
Lchr11
Lchr12
Lchr13
Lchr14
Lchr15
Lchr16
Lchr17
Lchr18
Lchr19
Lchr20
Lchr21
Lchr22
Features

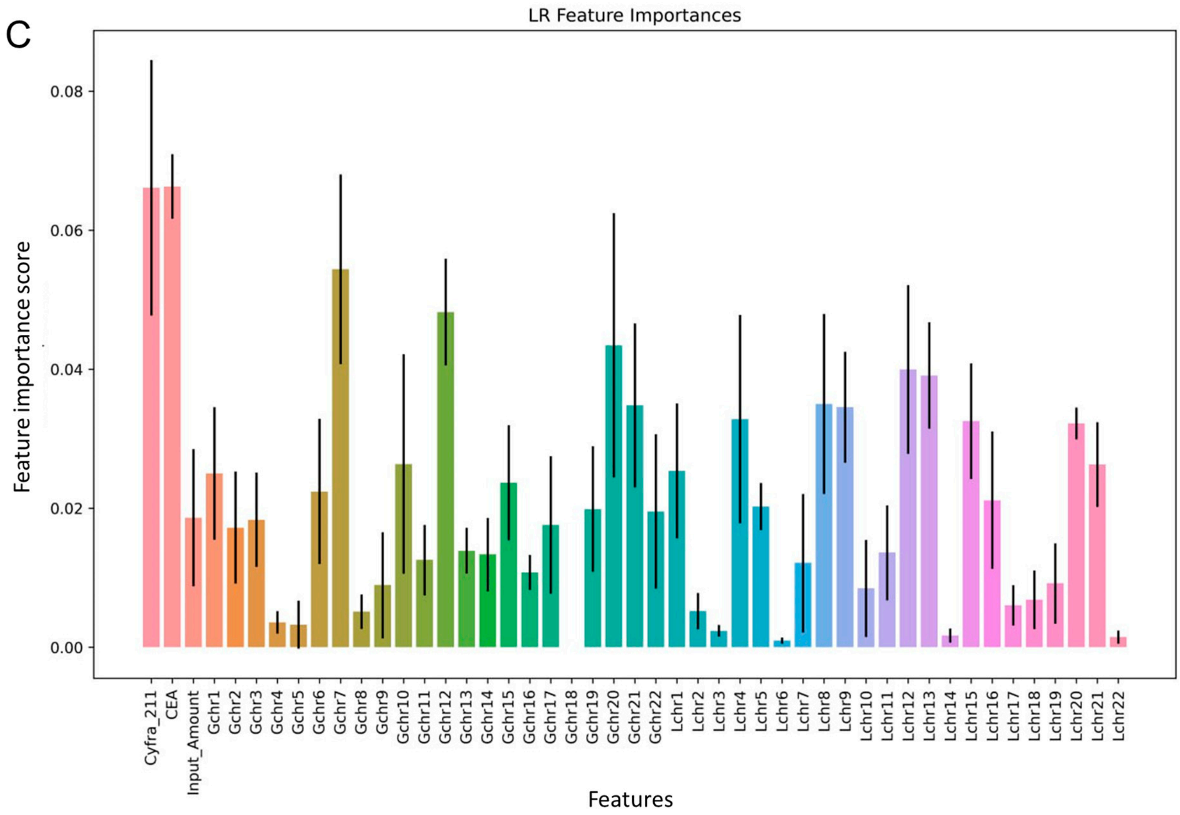

Supplementary Figure S2. Feature importance score of combined multi-omics data in AdaBoost model. Feature importance, encompassing all multi-omics data, for the AdaBoost (A), MLP (B), and LR (C) models in lung cancer prediction, quantified as the mean absolute values. The legend indicates the significance of the blood cancer markers (Cyfra21-1 and CEA), cfDNA concentration (Input_amount), and copy number variation (CNV, Gchr1~Lch22).
